# Psychometric properties of medication adherence instruments in cardiovascular diseases and type 2 diabetes mellitus: systematic review protocol

**DOI:** 10.1186/s13643-021-01755-w

**Published:** 2021-07-08

**Authors:** Henrique Ceretta Oliveira, Daisuke Hayashi Neto, Samantha Dalbosco Lins Carvalho, Rita de Cássia Lopes Barros, Mayza Luzia dos Santos Neves, Carla Renata Silva Andrechuk, Neusa Maria Costa Alexandre, Roberta Cunha Matheus Rodrigues

**Affiliations:** School of Nursing, University of Campinas (Unicamp), 126 Tessália Vieira de Camargo Street, Campinas, São Paulo, CEP: 13083-887 Brazil

**Keywords:** Medication adherence, Cardiovascular diseases, Diabetes mellitus, type 2, Patient reported outcome measures, Reproducibility of results, Psychometrics

## Abstract

**Background:**

The pharmacological treatment of cardiovascular diseases and type 2 diabetes mellitus reduces the risk of cardiovascular events.; however, most patients do not adhere to the treatment. There are several self-reported measures for assessing medication adherence. Identifying the instruments with the best psychometric evidence is essential for selecting an accurate measure. The aim of this study is to critically assess, compare and synthesize the quality of the measurement properties of patient-reported outcome measures to access medication adherence among patients with cardiovascular diseases and/or type 2 diabetes mellitus.

**Methods:**

This protocol is reported according to Preferred Reporting Items for Systematic Review and Meta-Analysis Protocols (PRISMA-P) and the COnsensus-based Standards for the selection of health Measurement INstruments (COSMIN) guidelines. The following databases will be searched: Web of Science, SCOPUS, PubMed, CINAHL, EMBASE, LILACS, PsycINFO and ProQuest.

**Discussion:**

This review will provide a detailed assessment of the measurement properties of self-reported medication adherence instruments in patients with cardiovascular diseases and/or type 2 diabetes mellitus to support clinical practice and research.

**Systematic review registration:**

PROSPERO CRD42019129109.

**Supplementary Information:**

The online version contains supplementary material available at 10.1186/s13643-021-01755-w.

## Background

Medication adherence represents an important challenge in the treatment of noncommunicable diseases (NCDs), as a considerable number of patients do not adhere to treatment [1]. It is associated with decreased mortality [2] and optimal quality of life [3].

Approximately 68% (38 million) of deaths worldwide were caused by NCDs, 50% of which were related to cardiovascular diseases (CVDs) and diabetes [4]. Poor drug adherence may result in clinical and psychosocial worsening of the disease, increased mortality, and increased healthcare costs [5].

Assessing and promoting medication adherence is paramount, considering the impact of medication regimens in improving glycemic control and decreasing the risk of cardiovascular events and mortality [5, 6].

Obtaining an accurate measurement of adherence has been as challenging as addressing the factors that lead to non-adherence because medication adherence behavior is complex, multifactorial, and influenced by different psychosocial variables such as motivation, self-efficacy, beliefs, and perceived barriers [7].

Several validated, patient-reported outcome measures (PROMs) are available in the literature to measure medication adherence among patients with different chronic diseases [8, 9]. The selection of an appropriate tool should consider its conceptual structure and the quality of its psychometric properties.

Some initiatives have been undertaken to evaluate the quality of the measurement properties of PROMs. In 2010, a taxonomy of terminologies and concepts related to measurement properties [10] and a checklist—COnsensus-based Standards for the selection of health Measurement INstruments (COSMIN) were created to evaluate the methodological quality of measurement properties studies [11].

Subsequent studies refined the checklist resulting in a guideline for systematic reviews on the measurement properties of PROMs [12]. This guideline proposes a combination of studies' methodological quality on measurement properties and the quality of the self-reported measurement itself.

There are several systematic reviews addressing the measurement properties of PROMs used to assess medication adherence in NCDs, but none of them have evaluated the quality of the measurement properties of medication adherence PROMs, according to COSMIN guidelines in patients with CVDs and/or type 2 diabetes mellitus (T2DM).

Therefore, this systematic review aims to critically assess, compare, and synthesize the PROMs' quality properties for medication adherence assessment among patients with CVDs and/or T2DM.

## Methods

This protocol was developed considering the Preferred Reporting Items for Systematic Review and Meta-Analysis Protocols (PRISMA-P) [13] (see checklist in Additional file 1) and in accordance with the COSMIN guideline for systematic reviews of PROMs [12]. The systematic review protocol was registered in the International Prospective Register of Systematic Reviews (PROSPERO CRD42019129109).

**Fig. 1:**
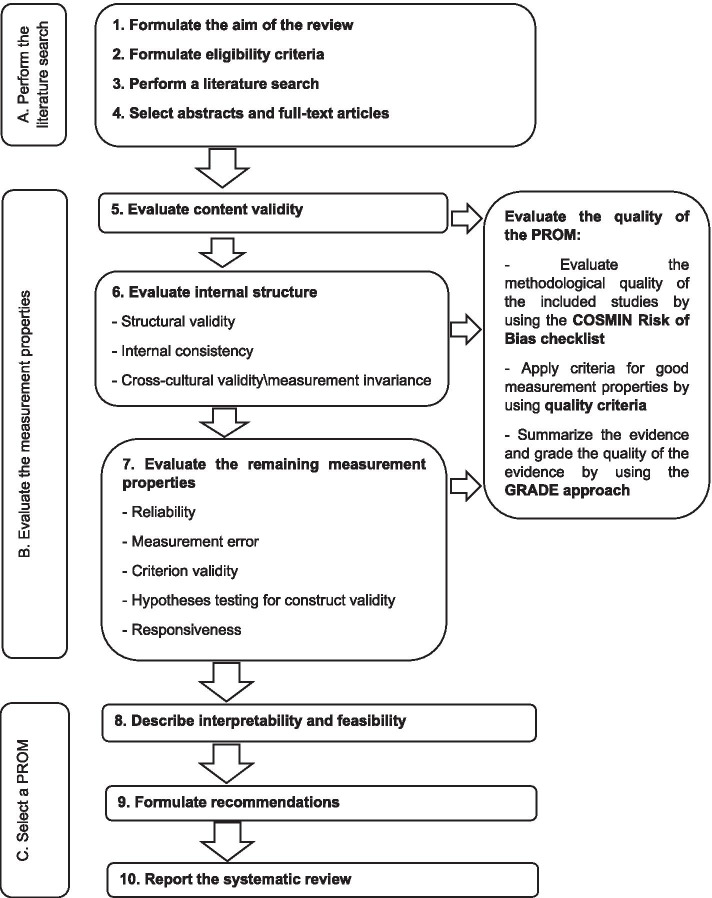
Flowchart of the steps necessary to perform a systematic review of PROMs. Source: Extracted from Prinsen et al., 2018, p. 1149. Note: PROM = Patient-reported outcome measures; COSMIN = Consensus-based Standards for the selection of Health Measurement Instruments; GRADE = Grading of Recommendations Assessment, Development, and Evaluation

According to the COSMIN guideline for systematic reviews of PROMs [12], ten stages divided into three parts are to be followed (Fig. 1):

- **Part A – Literature search:** which includes the definition of the review's objective, defining eligibility criteria, literature search, and selection of abstracts and full-text papers;
- **Part B – Assessment of the measurement properties:** content validity, internal structure, and remaining measurement properties. There are three sub-stages for each of these stages: studies' methodological quality, quality of results, and a summary of evidence and quality of evidence grading;
- **Part C – Selection of a PROM**: includes the description of interpretability and feasibility, recommendations, and the systematic review report.

### Eligibility criteria

This review will include:

- studies which aimed to develop or to culturally adapt a PROM to assess medication adherence among patients with a CVD and/or T2DM and who were older than 18 years of age, regardless of the language and date of publication;
- studies reporting the assessment of one or more properties of the PROMs.

This review will exclude:

- studies in which a PROM was used to measure an outcome (e.g., randomized clinical trials);
- studies in which a PROM was used to validate another measure;
- studies that evaluated the measurement properties of PROMs that aimed to evaluate the factors related to medication adherence (self-efficacy, beliefs, intention, etc.);
- studies that not provided sufficient information on outcomes of interest, even after contacting authors.

### Search strategy

The following databases were included: Web of Science, SCOPUS, PubMed, CINAHL, EMBASE, LILACS and PsycINFO. ProQuest was considered to search the gray literature. The search strategy considered terms related to cardiovascular disease, type 2 diabetes mellitus, PROMs, medication adherence and measurement properties. The second version of the search filter for measurement properties proposed by the COSMIN initiative was used in the search strategy [14]. The search strategy used in Pubmed is available in Additional file 2. Rayyan QCRI online software will be used to manage the references [15].

### Study selection

The process of study selection will be documented in accordance with the PRISMA flow diagram model [16]. Studies will be selected using four steps:

- (a) Exclusion of duplicates;
- (b) Titles and abstracts will be assessed according to eligibility criteria;
- (c) Full texts of potentially eligible studies identified in step 2 will be assessed to determine those that will be included in the review;
- (d) The list of references from the studies included in the review will be checked to identify other studies that haven’t been retrieved in previous searches.

### Anticipated results

The searches performed in July of 2020 resulted in a total of 41.886 papers. After the exclusion of duplicities (step a), a total of 27.060 papers will be evaluated according to their titles and abstracts (step b). The evaluation of titles and abstracts will be done independently by three pairs of reviewers. Therefore, each pair will evaluate 9.020 papers. Disagreements will be discussed with another reviewer until a consensus is obtained. The reviewers were trained and a pilot with 50 papers was performed to guarantee an inter-reviewer agreement.

### Data extraction

Data will be independently extracted by two reviewers. A standardized and pretested form will be used to extract data from the studies included in the review (characteristics of studies and information regarding PROMs) to assess the methodological quality of studies and summarize the evidence. Information will include: study design; sample size; participants' demographic and clinical characteristics (gender, age, disease, disease duration and number of taking medications); response rate; presence of conflicts of interest; funding; setting, country and language; PROMs number of items and domains; mode of administration; recall period; response options; range of scores; original language and available translations; number of studies evaluating the PROM; measurement properties (PROM development, content validity, structural validity, internal consistency, cross-cultural validity/measurement invariance, reliability, measurement error, criterion validity, hypothesis testing for construct validity and responsiveness); interpretability and feasibility; and information to assess the studies' methodological quality.

### Methodological quality/risk of bias

The studies' methodological quality will be assessed independently by two reviewers. COSMIN Risk of Bias checklist for systematic reviews of PROMs will be used to assess the methodological quality of the included studies [17, 18].

This checklist contains 116 items that assess the methodological quality of studies concerning aspects related to the measurement properties: PROMs development, content validity, structural validity, internal consistency, cross-cultural validity/measurement invariance, reliability, measurement error, criterion validity, hypotheses testing for construct validity, and responsiveness. The items can be rated as 'very good', 'adequate', 'doubtful', 'inadequate', or 'not applicable' (NA). An overall rating is assigned to each measurement property based on the worst scored item [18].

### Quality of the results concerning the measurement properties

The results will be assessed independently by two reviewers considering criteria for good measurement properties proposed by COSMIN [18]. Individual measurement properties of each assessed PROM will ultimately be classified as sufficient ( +), insufficient (-), inconsistent ( ±), or indeterminate (?) [12, 17, 18] (Table 1). If the data allow, a meta-analysis will be performed.

**Table 1:** Criteria for good measurement properties

Measurement property	Rating	Criteria
Structural validity	+	**CTT:** CFA: CFI or TLI or comparable measure > 0.95 OR RMSEA < 0.06 OR SRMR < 0.08 **IRT/Rasch:** No violation of unidimensionality: CFI or TLI or comparable measure > 0.95 OR RMSEA < 0.06 OR SRMR < 0.08 AND no violation of local independence: residual correlations among the items after controlling for the dominant factor < 0.20 OR Q3's < 0.37 AND no violation of monotonicity: adequate looking graphs OR item scalability > 0.30 AND adequate model fit IRT: χ2 > 0.001 Rasch: infit and outfit mean squares ≥ 0.5 and ≤ 1.5 OR Z-standardized values > − 2 and < 2
	?	CTT: not all information for ' + ' reported IRT/Rasch: model fit not reported
	-	Criteria for ' + ' not met
Internal consistency	+	At least low evidence for sufficient structural validity AND Cronbach's alpha(s) ≥ 0.70 for each unidimensional scale or subscale
	?	Criteria for “At least low evidence for sufficient structural validity” not met
	-	At least low evidence for sufficient structural validity AND Cronbach's alpha(s) < 0.70 for each unidimensional scale or subscale
Reliability	+	ICC or weighted Kappa ≥ 0.70
	?	ICC or weighted Kappa not reported
	-	ICC or weighted Kappa < 0.70
Measurement error	+	SDC or LoA < MIC
	?	MIC not defined
	-	SDC or LoA > MIC
Hypotheses testing for construct validity	+	The result is in accordance with the hypothesis
	?	No hypothesis defined (by the review team)
	-	The result is not in accordance with the hypothesis
Cross-cultural validity\measurement invariance	+	No important differences found between group factors (such as age, gender, language) in multiple group factor analysis OR no important DIF for group factors (McFadden's R^2^ < 0.02)
	?	No multiple group factor analysis OR DIF analysis performed
	-	Important differences between group factors OR DIF was found
Criterion validity	+	Correlation with gold standard ≥ 0.70 OR AUC ≥ 0.70
	?	Not all information for ' + ' reported
	-	Correlation with gold standard < 0.70 OR AUC < 0.70
Responsiveness	+	The result is in accordance with the hypothesis OR AUC ≥ 0.70
	?	No hypothesis defined (by the review team)
	-	The result is not in accordance with the hypothesis OR AUC < 0.70

Source: Extracted from Prinsen et al., 2018, p. 1152

Note: AUC = area under the curve; CFA = confirmatory factor analysis; CFI = comparative fit index; CTT = classical test theory; DIF = differential item functioning; ICC = intraclass correlation coefficient; IRT = item response theory; LoA = limits of agreement; MIC = minimal important change; RMSEA = root mean square error of approximation; SEM = standard error of measurement; SDC = smallest detectable change; SRMR = standardized root mean residuals; TLI = Tucker–Lewis index; “ + ” = sufficient; “ − ” = insufficient; “?” = indeterminate

### Quality of evidence

After summarizing the results, the quality of evidence of these results will be assessed considering an adaptation of the Grading of Recommendations Assessment, Development, and Evaluation (GRADE) proposed by the COSMIN initiative [12]. Evidence will be classified as high, moderate, poor, or very poor (Table 2).

**Table 2:** Definitions of quality levels

Quality level	Definition
High	We are very confident that the true measurement property lies close to that of the estimate of the measurement property
Moderate	We are moderately confident in the measurement property estimate: the true measurement property is likely to be close to the estimate of the measurement property, but there is a possibility that it is substantially different
Low	Our confidence in the measurement property estimate is limited: the true measurement property may be substantially different from the estimate of the measurement property
Very low	We have very little confidence in the measurement property estimate: the true measurement property is likely to be substantially different from the estimate of the measurement property

Source: Extracted from Prinsen et al., 2018, p. 1153

### Recommendations for selecting a PROM

The review's final stage will be the creation of recommendations to select the most appropriate PROM. PROMs will be classified into three categories:

- (a) PROMs that presented sufficient content validity and at least low quality of evidence for sufficient internal consistency;
- (b) PROMs that are not classified in categories (a) or (c);
- (c) PROMs that presented high quality of evidence for an insufficient measurement property.

A PROM that falls under category (a) means it is reliable and can be recommended. A PROM that falls under category (b) means it has the potential to be recommended, though further studies are needed to ensure its quality. A PROM classified under category (c) should not be recommended.

## Discussion

This review will provide a detailed assessment of the measurement properties of PROMs to measure medication adherence of patients with CVD and/or T2DM. Thus, based on this assessment, we expect to gather sufficient evidence regarding the most appropriate PROM to be used for these populations.

In the context of chronic diseases, studies that summarize knowledge and grading of evidence are essential, given the exponential increase of instruments, inconsistencies of validation methods and the importance of accurate measures for use in randomized clinical trials.

No recent systematic review addressing the quality of measurement properties of PROMs that assess medication adherence in the context of CVDs and T2DM was found. Therefore, this review's original contribution will be the use of current methodology with well-established quality as proposed by COSMIN [12].

Another strength of this review is the interdisciplinary nature of the team that designed this review protocol, composed of professionals from different areas of knowledge, including a statistician and researchers with expertise in the development, adaptation, and validation of self-reporting measures.

In summary, this protocol provides detailed information to plan a systematic review about the quality of measurement properties, which is a fundamental step to obtain clarity, transparency, and to ensure the reproducibility of the results of studies [19].

This review's results will support the recommendation of an instrument with the best psychometric evidence to measure medication adherence among patients with CVDs and/or T2DM. The systematic review is expected to facilitate the challenge of selecting an accurate self-reported measure of medication adherence for clinical and research use in this particular group of chronic diseases.

## Supplementary Information

**Additional file 1.**

**Additional file 2.**

## Data Availability

Not applicable.

## Abbreviations

CINAHL: Cumulative index to nursing and allied health literature
COSMIN: Consensus-based standards for the selection of health measurement instruments
CVDs: Cardiovascular diseases
EMBASE: Excerpta medica database
GRADE: Grading of recommendations assessment, development, and evaluation
LILACS: Literatura latino-americana e do Caribe em ciências da saúde
NCDs: Noncommunicable diseases
PRISMA-P: Preferred reporting items for systematic review and meta-analysis protocols
PROMs: Patient-reported outcome measures
PROSPERO: International prospective register of systematic reviews
T2DM: Type 2 diabetes mellitus
