# Supplementary material for: Psychometric properties of medication adherence instruments in cardiovascular diseases and type 2 diabetes mellitus: systematic review protocol
Source: Syst Rev. 2021 Jul 8;10:202. doi: 10.1186/s13643-021-01755-w (PMC8268561; doi:10.1186/s13643-021-01755-w)
Supplement: Supplementary file 2 — Additional file 2. [file 13643_2021_1755_MOESM2_ESM.pdf]

Search filter – Pubmed

Search	Query
**#1**	(instrumentation[sh] OR methods[sh] OR "Validation Studies"[pt] OR "Comparative Study"[pt] OR "psychometrics"[MeSH] OR psychometr*[tiab] OR clinimetr*[tw] OR clinometr*[tw] OR "outcome assessment (health care)"[MeSH] OR "outcome assessment"[tiab] OR "outcome measure*"[tw] OR "observer variation"[MeSH] OR "observer variation"[tiab] OR "Health Status Indicators"[Mesh] OR "reproducibility of results"[MeSH] OR reproducib*[tiab] OR "discriminant analysis"[MeSH] OR reliab*[tiab] OR unreliab*[tiab] OR valid*[tiab] OR "coefficient of variation"[tiab] OR coefficient[tiab] OR homogeneity[tiab] OR homogeneous[tiab] OR "internal consistency"[tiab] OR (cronbach*[tiab] AND (alpha[tiab] OR alphas[tiab])) OR (item[tiab] AND (correlation*[tiab] OR selection*[tiab] OR reduction*[tiab])) OR agreement[tw] OR precision[tw] OR imprecision[tw] OR "precise values"[tw] OR test-retest[tiab] OR (test[tiab] AND retest[tiab]) OR (reliab*[tiab] AND (test[tiab] OR retest[tiab])) OR stability[tiab] OR interrater[tiab] OR inter-rater[tiab] OR intrarater[tiab] OR intra-rater[tiab] OR intertester[tiab] OR inter-tester[tiab] OR intratester[tiab] OR intra-tester[tiab] OR interobserver[tiab] OR inter-observer[tiab] OR intraobserver[tiab] OR intra-observer[tiab] OR intertechnician[tiab] OR inter-technician[tiab] OR intratechnician[tiab] OR intra-technician[tiab] OR interexaminer[tiab] OR inter-examiner[tiab] OR intraexaminer[tiab] OR intra-examiner[tiab] OR interassay[tiab] OR inter-assay[tiab] OR intraassay[tiab] OR intra-assay[tiab] OR interindividual[tiab] OR inter-individual[tiab] OR intraindividual[tiab] OR intra-individual[tiab] OR interparticipant[tiab] OR inter-participant[tiab] OR intraparticipant[tiab] OR intra-participant[tiab] OR kappa[tiab] OR kappa's[tiab] OR kappas[tiab] OR repeatab*[tw] OR ((replicab*[tw] OR repeated[tw]) AND (measure[tw] OR measures[tw] OR findings[tw] OR result[tw] OR results[tw] OR test[tw] OR tests[tw])) OR generaliza*[tiab] OR generalisa*[tiab] OR concordance[tiab] OR (intraclass[tiab] AND correlation*[tiab]) OR discriminative[tiab] OR "known group"[tiab] OR "factor analysis"[tiab] OR "factor analyses"[tiab] OR "factor structure"[tiab] OR "factor structures"[tiab] OR dimension*[tiab] OR subscale*[tiab] OR (multitrait[tiab] AND scaling[tiab] AND (analysis[tiab] OR analyses[tiab])) OR "item discriminant"[tiab] OR "interscale correlation*"[tiab] OR error[tiab] OR errors[tiab] OR "individual variability"[tiab] OR "interval variability"[tiab] OR "rate variability"[tiab] OR (variability[tiab] AND (analysis[tiab] OR values[tiab])) OR (uncertainty[tiab] AND (measurement[tiab] OR measuring[tiab])) OR "standard error of measurement"[tiab] OR sensitiv*[tiab] OR responsive*[tiab] OR (limit[tiab] AND detection[tiab]) OR "minimal detectable concentration"[tiab] OR interpretab*[tiab] OR ((minimal[tiab] OR minimally[tiab] OR clinical[tiab] OR clinically[tiab]) AND (important[tiab] OR significant[tiab] OR detectable[tiab]) AND (change[tiab] OR difference[tiab])) OR (small*[tiab] AND (real[tiab] OR detectable[tiab]) AND (change[tiab] OR difference[tiab])) OR "meaningful change"[tiab] OR "ceiling effect"[tiab] OR "floor effect"[tiab] OR "Item response model"[tiab] OR IRT[tiab] OR Rasch[tiab] OR "Differential item functioning"[tiab] OR DIF[tiab] OR "computer adaptive testing"[tiab] OR "item bank"[tiab] OR "cross-cultural equivalence"[tiab])
**#2**	((Surveys and Questionnaires[MeSH Terms]) OR ("Surveys and Questionnaires"[Title/Abstract])) OR (Survey Methods[MeSH Terms])) OR ("Survey Methods"[Title/Abstract])) OR (Questionnaires and Surveys[MeSH Terms])) OR ("Questionnaires and Surveys"[Title/Abstract])) OR (Methods, Survey[MeSH Terms])) OR ("Methods, Survey"[Title/Abstract])) OR (Survey Method[MeSH Terms])) OR ("Survey Method"[Title/Abstract])) OR (Methodology, Survey[MeSH Terms])) OR ("Methodology, Survey"[Title/Abstract])) OR (Survey Methodology[MeSH Terms])) OR ("Survey Methodology"[Title/Abstract])) OR (Community Surveys[MeSH Terms])) OR ("Community Surveys"[Title/Abstract])) OR (Community Survey[MeSH Terms])) OR ("Community Survey"[Title/Abstract])) OR (Survey, Community[MeSH Terms])) OR ("Survey, Community"[Title/Abstract])) OR (Surveys, Community[MeSH Terms])) OR ("Surveys, Community"[Title/Abstract])) OR (Repeated Rounds of Survey[MeSH Terms])) OR ("Repeated Rounds of Survey"[Title/Abstract])) OR (Surveys[MeSH Terms])) OR (Surveys[Title/Abstract])) OR (Survey[MeSH Terms])) OR (Survey[Title/Abstract])) OR (Questionnaire Design[MeSH Terms])) OR ("Questionnaire Design"[Title/Abstract])) OR (Design, Questionnaire[MeSH Terms])) OR ("Design, Questionnaire"[Title/Abstract])) OR (Designs, Questionnaire[MeSH Terms])) OR ("Designs, Questionnaire"[Title/Abstract])) OR (Questionnaire Designs[MeSH Terms])) OR ("Questionnaire Designs"[Title/Abstract])) OR (Baseline Survey[MeSH Terms])) OR ("Baseline Survey"[Title/Abstract])) OR (Baseline Surveys[MeSH Terms])) OR ("Baseline Surveys"[Title/Abstract])) OR (Survey, Baseline[MeSH Terms])) OR ("Survey, Baseline"[Title/Abstract])) OR (Surveys, Baseline[MeSH Terms])) OR ("Surveys,

	Baseline"[Title/Abstract])) OR (Respondents[MeSH Terms])) OR (Respondents[Title/Abstract])) OR (Respondent[MeSH Terms])) OR (Respondent[Title/Abstract])) OR (Randomized Response Technique[MeSH Terms])) OR ("Randomized Response Technique"[Title/Abstract])) OR (Randomized Response Techniques[MeSH Terms])) OR ("Randomized Response Techniques"[Title/Abstract])) OR (Response Technique, Randomized[MeSH Terms])) OR ("Response Technique, Randomized"[Title/Abstract])) OR (Response Techniques, Randomized[MeSH Terms])) OR ("Response Techniques, Randomized"[Title/Abstract])) OR (Techniques, Randomized Response[MeSH Terms])) OR ("Techniques, Randomized Response"[Title/Abstract])) OR (Questionnaires[MeSH Terms])) OR (Questionnaires[Title/Abstract])) OR (Questionnaire[MeSH Terms])) OR (Questionnaire[Title/Abstract])) OR (Nonrespondents[MeSH Terms])) OR (Nonrespondents[Title/Abstract])) OR (Nonrespondent[MeSH Terms])) OR (Nonrespondent[Title/Abstract])) OR ((((((((((((((Patient Reported Outcome Measures[MeSH Terms]) OR ("Patient Reported Outcome Measures"[Title/Abstract])) OR (Patient Reported Outcomes[MeSH Terms])) OR ("Patient Reported Outcomes"[Title/Abstract])) OR (Outcome, Patient Reported[MeSH Terms])) OR ("Outcome, Patient Reported"[Title/Abstract])) OR (Outcomes, Patient Reported[MeSH Terms])) OR ("Outcomes, Patient Reported"[Title/Abstract])) OR (Reported Outcome, Patient[MeSH Terms])) OR ("Reported Outcome, Patient"[Title/Abstract])) OR (Reported Outcomes, Patient[MeSH Terms])) OR ("Reported Outcomes, Patient"[Title/Abstract])) OR (Patient Reported Outcome[MeSH Terms])) OR ("Patient Reported Outcome"[Title/Abstract]))) OR ((((((((Self Report[MeSH Terms]) OR ("Self Report"[Title/Abstract])) OR (Report, Self[MeSH Terms])) OR ("Report, Self"[Title/Abstract])) OR (Reports, Self[MeSH Terms])) OR ("Reports, Self"[Title/Abstract])) OR (Self Reports[MeSH Terms])) OR ("Self Reports"[Title/Abstract]))) OR (index)) OR (indices)) OR ("self-report")) OR ("self-report measures")) OR ("assessment tools")) OR ("patient-reported outcome")) OR ("measurement scale")) OR ("health measurement scale")) OR ("measure health outcomes")) OR (instrument)) OR ("measurement instrument")) OR (scale)) OR (measure)) OR (tool)
**#3**	(((((((((((((((((((((((((((((((((((((Medication Adherence[MeSH Terms]) OR "Medication Adherence"[Title/Abstract]) OR Adherence, Medication[MeSH Terms]) OR "Adherence, Medication"[Title/Abstract]) OR Medication Nonadherence[MeSH Terms]) OR "Medication Nonadherence"[Title/Abstract]) OR Nonadherence, Medication[MeSH Terms]) OR "Nonadherence, Medication"[Title/Abstract]) OR Medication Noncompliance[MeSH Terms]) OR "Medication Noncompliance"[Title/Abstract]) OR Noncompliance, Medication[MeSH Terms]) OR "Noncompliance, Medication"[Title/Abstract]) OR Medication Non-Adherence[MeSH Terms]) OR "Medication Non-Adherence"[Title/Abstract]) OR Medication Non Adherence[MeSH Terms]) OR "Medication Non Adherence"[Title/Abstract]) OR Non-Adherence, Medication[MeSH Terms]) OR "Non-Adherence, Medication"[Title/Abstract]) OR Medication Persistence[MeSH Terms]) OR "Medication Persistence"[Title/Abstract]) OR Persistence, Medication[MeSH Terms]) OR "Persistence, Medication"[Title/Abstract]) OR Medication Compliance[MeSH Terms]) OR "Medication Compliance"[Title/Abstract]) OR Compliance, Medication[MeSH Terms]) OR "Compliance, Medication"[Title/Abstract]) OR Medication Non-Compliance[MeSH Terms]) OR "Medication Non-Compliance"[Title/Abstract]) OR Medication Non Compliance[MeSH Terms]) OR "Medication Non Compliance"[Title/Abstract]) OR Non-Compliance, Medication[MeSH Terms]) OR "Non-Compliance, Medication"[Title/Abstract]) OR ((Patient Compliance[MeSH Terms]) OR "Patient Compliance"[Title/Abstract]) OR Compliance, Patient[MeSH Terms]) OR "Compliance, Patient"[Title/Abstract]) OR Patient Adherence[MeSH Terms]) OR "Patient Adherence"[Title/Abstract]) OR Adherence, Patient[MeSH Terms]) OR "Adherence, Patient"[Title/Abstract]) OR Patient Cooperation[MeSH Terms]) OR "Patient Cooperation"[Title/Abstract]) OR Cooperation, Patient[MeSH Terms]) OR "Cooperation, Patient"[Title/Abstract]) OR Patient Non-Compliance[MeSH Terms]) OR "Patient Non-Compliance"[Title/Abstract]) OR Non-Compliance, Patient[MeSH Terms]) OR "Non-Compliance, Patient"[Title/Abstract]) OR Patient Non Compliance[MeSH Terms]) OR "Patient Non Compliance"[Title/Abstract]) OR Patient Nonadherence[MeSH Terms]) OR "Patient Nonadherence"[Title/Abstract]) OR Nonadherence, Patient[MeSH Terms]) OR "Nonadherence, Patient"[Title/Abstract]) OR Patient Noncompliance[MeSH Terms]) OR "Patient Noncompliance"[Title/Abstract]) OR Noncompliance, Patient[MeSH Terms]) OR "Noncompliance, Patient"[Title/Abstract]) OR Patient Non-Adherence[MeSH Terms]) OR "Patient Non-Adherence"[Title/Abstract]) OR Non-Adherence, Patient[MeSH Terms]) OR "Non-Adherence, Patient"[Title/Abstract]) OR Patient Non Adherence[MeSH Terms]) OR "Patient Non Adherence"[Title/Abstract]) OR Treatment Compliance[MeSH Terms]) OR "Treatment

	Compliance"[Title/Abstract]) OR Compliance, Treatment[MeSH Terms]) OR "Compliance, Treatment"[Title/Abstract]) OR Treatment Compliances[MeSH Terms]) OR "Treatment Compliances"[Title/Abstract]) OR Therapeutic Compliance[MeSH Terms]) OR "Therapeutic Compliance"[Title/Abstract]) OR Compliance, Therapeutic[MeSH Terms]) OR "Compliance, Therapeutic"[Title/Abstract]) OR Compliances, Therapeutic[MeSH Terms]) OR "Compliances, Therapeutic"[Title/Abstract]) OR Therapeutic Compliances[MeSH Terms]) OR "Therapeutic Compliances"[Title/Abstract])) OR ("drug adherence")) OR ("drug compliance")) OR ("taking medication")) OR ("medication initiation")) OR ("medication implementation")
**#4**	(((((((((((((((((((((Cardiovascular Diseases[MeSH Terms]) OR "Cardiovascular Diseases"[Title/Abstract]) OR Cardiovascular Disease[MeSH Terms]) OR "Cardiovascular Disease"[Title/Abstract]) OR Disease, Cardiovascular[MeSH Terms]) OR "Diseases, Cardiovascular"[Title/Abstract]) OR Diseases, Cardiovascular[MeSH Terms]) OR "Diseases, Cardiovascular"[Title/Abstract])) OR ((((((((((((((((Heart Diseases[MeSH Terms]) OR "Heart Diseases"[Title/Abstract]) OR Disease, Heart[MeSH Terms]) OR "Disease, Heart"[Title/Abstract]) OR Diseases, Heart[MeSH Terms]) OR "Diseases, Heart"[Title/Abstract]) OR Heart Disease[MeSH Terms]) OR "Heart Disease"[Title/Abstract]) OR Cardiac Diseases[MeSH Terms]) OR "Cardiac Diseases"[Title/Abstract]) OR Cardiac Disease[MeSH Terms]) OR "Cardiac Disease"[Title/Abstract]) OR Disease, Cardiac[MeSH Terms]) OR "Disease, Cardiac"[Title/Abstract]) OR Diseases, Cardiac[MeSH Terms]) OR "Diseases, Cardiac"[Title/Abstract])) OR ((((((((Vascular Diseases[MeSH Terms]) OR "Vascular Diseases"[Title/Abstract]) OR Disease, Vascular[MeSH Terms]) OR "Disease, Vascular"[Title/Abstract]) OR Diseases, Vascular[MeSH Terms]) OR "Diseases, Vascular"[Title/Abstract]) OR Vascular Disease[MeSH Terms]) OR "Vascular Disease"[Title/Abstract])) OR ((((((((((((((((((((Coronary Disease[MeSH Terms]) OR "Coronary Disease"[Title/Abstract]) OR Coronary Diseases[MeSH Terms]) OR "Coronary Diseases"[Title/Abstract]) OR Disease, Coronary[MeSH Terms]) OR "Disease, Coronary"[Title/Abstract]) OR Diseases, Coronary[MeSH Terms]) OR "Diseases, Coronary"[Title/Abstract]) OR Coronary Heart Disease[MeSH Terms]) OR "Coronary Heart Disease"[Title/Abstract]) OR Coronary Heart Diseases[MeSH Terms]) OR "Coronary Heart Diseases"[Title/Abstract]) OR Disease, Coronary Heart[MeSH Terms]) OR "Disease, Coronary Heart"[Title/Abstract]) OR Diseases, Coronary Heart[MeSH Terms]) OR "Diseases, Coronary Heart"[Title/Abstract]) OR Heart Disease, Coronary[MeSH Terms]) OR "Heart Disease, Coronary"[Title/Abstract]) OR Heart Diseases, Coronary[MeSH Terms]) OR "Heart Diseases, Coronary"[Title/Abstract])) OR ((Cerebrovascular Disorders[MeSH Terms]) OR "Cerebrovascular Disorders"[Title/Abstract]) OR Cerebrovascular Disorder[MeSH Terms]) OR "Cerebrovascular Disorder"[Title/Abstract]) OR Vascular Diseases, Intracranial[MeSH Terms]) OR "Vascular Diseases, Intracranial"[Title/Abstract]) OR Intracranial Vascular Disease[MeSH Terms]) OR "Intracranial Vascular Disease"[Title/Abstract]) OR Intracranial Vascular Diseases[MeSH Terms]) OR "Intracranial Vascular Diseases"[Title/Abstract]) OR Vascular Disease, Intracranial[MeSH Terms]) OR "Vascular Disease, Intracranial"[Title/Abstract]) OR Intracranial Vascular Disorders[MeSH Terms]) OR "Intracranial Vascular Disorders"[Title/Abstract]) OR Intracranial Vascular Disorder[MeSH Terms]) OR "Intracranial Vascular Disorder"[Title/Abstract]) OR Vascular Disorder, Intracranial[MeSH Terms]) OR "Vascular Disorder, Intracranial"[Title/Abstract]) OR Vascular Disorders, Intracranial[MeSH Terms]) OR "Vascular Disorders, Intracranial"[Title/Abstract]) OR Cerebrovascular Diseases[MeSH Terms]) OR "Cerebrovascular Diseases"[Title/Abstract]) OR Cerebrovascular Disease[MeSH Terms]) OR "Cerebrovascular Disease"[Title/Abstract]) OR Disease, Cerebrovascular[MeSH Terms]) OR "Disease, Cerebrovascular"[Title/Abstract]) OR Diseases, Cerebrovascular[MeSH Terms]) OR "Diseases, Cerebrovascular"[Title/Abstract]) OR Brain Vascular Disorders[MeSH Terms]) OR "Brain Vascular Disorders"[Title/Abstract]) OR Brain Vascular Disorder[MeSH Terms]) OR "Brain Vascular Disorder"[Title/Abstract]) OR Vascular Disorder, Brain[MeSH Terms]) OR "Vascular Disorder, Brain"[Title/Abstract]) OR Vascular Disorders, Brain[MeSH Terms]) OR "Vascular Disorders, Brain"[Title/Abstract]) OR Cerebrovascular Occlusion[MeSH Terms]) OR "Cerebrovascular Occlusion"[Title/Abstract]) OR Cerebrovascular Occlusions[MeSH Terms]) OR "Cerebrovascular Occlusions"[Title/Abstract]) OR Occlusion, Cerebrovascular[MeSH Terms]) OR "Occlusion, Cerebrovascular"[Title/Abstract]) OR Occlusions, Cerebrovascular[MeSH Terms]) OR "Occlusions, Cerebrovascular"[Title/Abstract]) OR Cerebrovascular Insufficiency[MeSH Terms]) OR "Cerebrovascular Insufficiency"[Title/Abstract]) OR Cerebrovascular Insufficiencies[MeSH Terms]) OR "Cerebrovascular Insufficiencies"[Title/Abstract]) OR Insufficiencies, Cerebrovascular[MeSH Terms]) OR "Insufficiencies, Cerebrovascular"[Title/Abstract]) OR Insufficiency, Cerebrovascular[MeSH Terms]) OR "Insufficiency, Cerebrovascular"[Title/Abstract])) OR ((((((((((((((((((((((((Peripheral Arterial Disease[MeSH Terms]) OR

	"Peripheral Arterial Disease"[Title/Abstract]) OR Arterial Disease, Peripheral[MeSH Terms]) OR "Arterial Disease, Peripheral"[Title/Abstract]) OR Arterial Diseases, Peripheral[MeSH Terms]) OR "Arterial Diseases, Peripheral"[Title/Abstract]) OR Disease, Peripheral Arterial[MeSH Terms]) OR "Disease, Peripheral Arterial"[Title/Abstract]) OR Diseases, Peripheral Arterial[MeSH Terms]) OR "Diseases, Peripheral Arterial"[Title/Abstract]) OR Peripheral Arterial Diseases[MeSH Terms]) OR "Peripheral Arterial Diseases"[Title/Abstract]) OR Peripheral Artery Disease[MeSH Terms]) OR "Peripheral Artery Disease"[Title/Abstract]) OR Artery Disease, Peripheral[MeSH Terms]) OR "Artery Disease, Peripheral"[Title/Abstract]) OR Artery Diseases, Peripheral[MeSH Terms]) OR "Artery Diseases, Peripheral"[Title/Abstract]) OR Disease, Peripheral Artery[MeSH Terms]) OR "Disease, Peripheral Artery"[Title/Abstract]) OR Diseases, Peripheral Artery[MeSH Terms]) OR "Diseases, Peripheral Artery"[Title/Abstract]) OR Peripheral Artery Diseases[MeSH Terms]) OR "Peripheral Artery Diseases"[Title/Abstract])) OR ((((((((((((((((((((((Rheumatic Heart Disease[MeSH Terms]) OR "Rheumatic Heart Disease"[Title/Abstract]) OR Disease, Rheumatic Heart[MeSH Terms]) OR "Disease, Rheumatic Heart"[Title/Abstract]) OR Diseases, Rheumatic Heart[MeSH Terms]) OR "Diseases, Rheumatic Heart"[Title/Abstract]) OR Heart Disease, Rheumatic[MeSH Terms]) OR "Heart Disease, Rheumatic"[Title/Abstract]) OR Heart Diseases, Rheumatic[MeSH Terms]) OR "Heart Diseases, Rheumatic"[Title/Abstract]) OR Rheumatic Heart Diseases[MeSH Terms]) OR "Rheumatic Heart Diseases"[Title/Abstract]) OR Bouillaud Disease[MeSH Terms]) OR "Bouillaud Disease"[Title/Abstract]) OR Disease, Bouillaud[MeSH Terms]) OR "Disease, Bouillaud"[Title/Abstract]) OR Bouillaud's Disease[MeSH Terms]) OR "Bouillaud's Disease"[Title/Abstract]) OR Bouillauds Disease[MeSH Terms]) OR "Bouillauds Disease"[Title/Abstract]) OR Disease, Bouillaud's[MeSH Terms]) OR "Disease, Bouillaud's"[Title/Abstract])) OR ((Venous Thrombosis[MeSH Terms]) OR "Venous Thrombosis"[Title/Abstract]) OR Phlebothrombosis[MeSH Terms]) OR Phlebothrombosis[Title/Abstract]) OR Phlebothromboses[MeSH Terms]) OR Phlebothromboses[Title/Abstract]) OR Thrombosis, Venous[MeSH Terms]) OR "Thrombosis, Venous"[Title/Abstract]) OR Thromboses, Venous[MeSH Terms]) OR "Thromboses, Venous"[Title/Abstract]) OR Venous Thromboses[MeSH Terms]) OR "Venous Thromboses"[Title/Abstract]) OR Deep Vein Thrombosis[MeSH Terms]) OR "Deep Vein Thrombosis"[Title/Abstract]) OR Deep Vein Thromboses[MeSH Terms]) OR "Deep Vein Thromboses"[Title/Abstract]) OR Thromboses, Deep Vein[MeSH Terms]) OR "Thromboses, Deep Vein"[Title/Abstract]) OR Vein Thromboses, Deep[MeSH Terms]) OR "Vein Thromboses, Deep"[Title/Abstract]) OR Vein Thrombosis, Deep[MeSH Terms]) OR "Vein Thrombosis, Deep"[Title/Abstract]) OR Deep-Venous Thrombosis[MeSH Terms]) OR "Deep-Venous Thrombosis"[Title/Abstract]) OR Deep-Venous Thromboses[MeSH Terms]) OR "Deep-Venous Thromboses"[Title/Abstract]) OR Thromboses, Deep-Venous[MeSH Terms]) OR "Thromboses, Deep-Venous"[Title/Abstract]) OR Thrombosis, Deep-Venous[MeSH Terms]) OR "Thrombosis, Deep-Venous"[Title/Abstract]) OR Deep-Vein Thrombosis[MeSH Terms]) OR "Deep-Vein Thrombosis"[Title/Abstract]) OR Deep-Vein Thromboses[MeSH Terms]) OR "Deep-Vein Thromboses"[Title/Abstract]) OR Thromboses, Deep-Vein[MeSH Terms]) OR "Thromboses, Deep-Vein"[Title/Abstract]) OR Thrombosis, Deep-Vein[MeSH Terms]) OR "Thrombosis, Deep-Vein"[Title/Abstract]) OR Thrombosis, Deep Vein[MeSH Terms]) OR "Thrombosis, Deep Vein"[Title/Abstract]) OR Deep Venous Thrombosis[MeSH Terms]) OR "Deep Venous Thrombosis"[Title/Abstract]) OR Deep Venous Thromboses[MeSH Terms]) OR "Deep Venous Thromboses"[Title/Abstract]) OR Thromboses, Deep Venous[MeSH Terms]) OR "Thromboses, Deep Venous"[Title/Abstract]) OR Thrombosis, Deep Venous[MeSH Terms]) OR "Thrombosis, Deep Venous"[Title/Abstract]) OR Venous Thromboses, Deep[MeSH Terms]) OR "Venous Thromboses, Deep"[Title/Abstract]) OR Venous Thrombosis, Deep[MeSH Terms]) OR "Venous Thrombosis, Deep"[Title/Abstract])) OR ((((((((((((((((Pulmonary Embolism[MeSH Terms]) OR "Pulmonary Embolism"[Title/Abstract]) OR Pulmonary Embolisms[MeSH Terms]) OR "Pulmonary Embolisms"[Title/Abstract]) OR Embolism, Pulmonary[MeSH Terms]) OR "Embolism, Pulmonary"[Title/Abstract]) OR Embolisms, Pulmonary[MeSH Terms]) OR "Embolisms, Pulmonary"[Title/Abstract]) OR Pulmonary Thromboembolisms[MeSH Terms]) OR "Pulmonary Thromboembolisms"[Title/Abstract]) OR Pulmonary Thromboembolism[MeSH Terms]) OR "Pulmonary Thromboembolism"[Title/Abstract]) OR Thromboembolism, Pulmonary[MeSH Terms]) OR "Thromboembolism, Pulmonary"[Title/Abstract]) OR Thromboembolisms, Pulmonary[MeSH Terms]) OR "Thromboembolisms, Pulmonary"[Title/Abstract])) OR (((Stroke[MeSH Terms]) OR Stroke[Title/Abstract]) OR Strokes[MeSH Terms]) OR Strokes[Title/Abstract]) OR Cerebrovascular Accident[MeSH Terms]) OR

	"Cerebrovascular Accident"[Title/Abstract]) OR Cerebrovascular Accidents[MeSH Terms]) OR "Cerebrovascular Accidents"[Title/Abstract]) OR CVA (Cerebrovascular Accident)[MeSH Terms]) OR "CVA (Cerebrovascular Accident)"[Title/Abstract]) OR CVAs (Cerebrovascular Accident)[MeSH Terms]) OR "CVAs (Cerebrovascular Accident)"[Title/Abstract]) OR Cerebrovascular Apoplexy[MeSH Terms]) OR "Cerebrovascular Apoplexy"[Title/Abstract]) OR Apoplexy, Cerebrovascular[MeSH Terms]) OR "Apoplexy, Cerebrovascular"[Title/Abstract]) OR Vascular Accident, Brain[MeSH Terms]) OR "Vascular Accident, Brain"[Title/Abstract]) OR Brain Vascular Accident[MeSH Terms]) OR "Brain Vascular Accident"[Title/Abstract]) OR Brain Vascular Accidents[MeSH Terms]) OR "Brain Vascular Accidents"[Title/Abstract]) OR Vascular Accidents, Brain[MeSH Terms]) OR "Vascular Accidents, Brain"[Title/Abstract]) OR Cerebrovascular Stroke[MeSH Terms]) OR "Cerebrovascular Stroke"[Title/Abstract]) OR Cerebrovascular Strokes[MeSH Terms]) OR "Cerebrovascular Strokes"[Title/Abstract]) OR Stroke, Cerebrovascular[MeSH Terms]) OR "Stroke, Cerebrovascular"[Title/Abstract]) OR Strokes, Cerebrovascular[MeSH Terms]) OR "Strokes, Cerebrovascular"[Title/Abstract]) OR Apoplexy[MeSH Terms]) OR Apoplexy[Title/Abstract]) OR Cerebral Stroke[MeSH Terms]) OR "Cerebral Stroke"[Title/Abstract]) OR Cerebral Strokes[MeSH Terms]) OR "Cerebral Strokes"[Title/Abstract]) OR Stroke, Cerebral[MeSH Terms]) OR "Stroke, Cerebral"[Title/Abstract]) OR Strokes, Cerebral[MeSH Terms]) OR "Strokes, Cerebral"[Title/Abstract]) OR Stroke, Acute[MeSH Terms]) OR "Stroke, Acute"[Title/Abstract]) OR Acute Stroke[MeSH Terms]) OR "Acute Stroke"[Title/Abstract]) OR Acute Strokes[MeSH Terms]) OR "Acute Strokes"[Title/Abstract]) OR Strokes, Acute[MeSH Terms]) OR "Strokes, Acute"[Title/Abstract]) OR Cerebrovascular Accident, Acute[MeSH Terms]) OR "Cerebrovascular Accident, Acute"[Title/Abstract]) OR Acute Cerebrovascular Accident[MeSH Terms]) OR "Acute Cerebrovascular Accident"[Title/Abstract]) OR Acute Cerebrovascular Accidents[MeSH Terms]) OR "Acute Cerebrovascular Accidents"[Title/Abstract]) OR Cerebrovascular Accidents, Acute[MeSH Terms]) OR "Cerebrovascular Accidents, Acute"[Title/Abstract])) OR ((((((((((Hypertension[MeSH Terms]) OR Hypertension[Title/Abstract]) OR Blood Pressure, High[MeSH Terms]) OR "Blood Pressure, High"[Title/Abstract]) OR Blood Pressures, High[MeSH Terms]) OR "Blood Pressures, High"[Title/Abstract]) OR High Blood Pressure[MeSH Terms]) OR "High Blood Pressure"[Title/Abstract]) OR High Blood Pressures[MeSH Terms]) OR "High Blood Pressures"[Title/Abstract])) OR ((((((((((((((((((((((((((((((Myocardial Infarction[MeSH Terms]) OR "Myocardial Infarction"[Title/Abstract]) OR Infarction, Myocardial[MeSH Terms]) OR "Infarction, Myocardial"[Title/Abstract]) OR Infarctions, Myocardial[MeSH Terms]) OR "Infarctions, Myocardial"[Title/Abstract]) OR Myocardial Infarctions[MeSH Terms]) OR "Myocardial Infarctions"[Title/Abstract]) OR Cardiovascular Stroke[MeSH Terms]) OR "Cardiovascular Stroke"[Title/Abstract]) OR Cardiovascular Strokes[MeSH Terms]) OR "Cardiovascular Strokes"[Title/Abstract]) OR Stroke, Cardiovascular[MeSH Terms]) OR "Stroke, Cardiovascular"[Title/Abstract]) OR Strokes, Cardiovascular[MeSH Terms]) OR "Strokes, Cardiovascular"[Title/Abstract]) OR Heart Attack[MeSH Terms]) OR "Heart Attack"[Title/Abstract]) OR Heart Attacks[MeSH Terms]) OR "Heart Attacks"[Title/Abstract]) OR Myocardial Infarct[MeSH Terms]) OR "Myocardial Infarct"[Title/Abstract]) OR Infarct, Myocardial[MeSH Terms]) OR "Infarct, Myocardial"[Title/Abstract]) OR Infarcts, Myocardial[MeSH Terms]) OR "Infarcts, Myocardial"[Title/Abstract]) OR Myocardial Infarcts[MeSH Terms]) OR "Myocardial Infarcts"[Title/Abstract])) OR ((((((((((((((((((((((((((((((Heart Failure[MeSH Terms]) OR "Heart Failure"[Title/Abstract]) OR Cardiac Failure[MeSH Terms]) OR "Cardiac Failure"[Title/Abstract]) OR Heart Decompensation[MeSH Terms]) OR "Heart Decompensation"[Title/Abstract]) OR Decompensation, Heart[MeSH Terms]) OR "Decompensation, Heart"[Title/Abstract]) OR Heart Failure, Right-Sided[MeSH Terms]) OR "Heart Failure, Right-Sided"[Title/Abstract]) OR Heart Failure, Right Sided[MeSH Terms]) OR "Heart Failure, Right Sided"[Title/Abstract]) OR Right-Sided Heart Failure[MeSH Terms]) OR "Right-Sided Heart Failure"[Title/Abstract]) OR Right Sided Heart Failure[MeSH Terms]) OR "Right Sided Heart Failure"[Title/Abstract]) OR Myocardial Failure[MeSH Terms]) OR "Myocardial Failure"[Title/Abstract]) OR Congestive Heart Failure[MeSH Terms]) OR "Congestive Heart Failure"[Title/Abstract]) OR Heart Failure, Congestive[MeSH Terms]) OR "Heart Failure, Congestive"[Title/Abstract]) OR Heart Failure, Left-Sided[MeSH Terms]) OR "Heart Failure, Left-Sided"[Title/Abstract]) OR Heart Failure, Left Sided[MeSH Terms]) OR "Heart Failure, Left Sided"[Title/Abstract]) OR Left-Sided Heart Failure[MeSH Terms]) OR "Left-Sided Heart Failure"[Title/Abstract]) OR Left Sided Heart Failure[MeSH Terms]) OR "Left Sided Heart Failure"[Title/Abstract])) OR (((Diabetes Mellitus, Type 2[MeSH Terms]) OR "Diabetes Mellitus, Type 2"[Title/Abstract]) OR Diabetes Mellitus, Noninsulin-Dependent[MeSH Terms]) OR "Diabetes Mellitus, Noninsulin-Dependent"[Title/Abstract]) OR Diabetes

	Mellitus, Ketosis-Resistant[MeSH Terms]) OR "Diabetes Mellitus, Ketosis-Resistant"[Title/Abstract]) OR Diabetes Mellitus, Ketosis Resistant[MeSH Terms]) OR "Diabetes Mellitus, Ketosis Resistant"[Title/Abstract]) OR Ketosis-Resistant Diabetes Mellitus[MeSH Terms]) OR "Ketosis-Resistant Diabetes Mellitus"[Title/Abstract]) OR Diabetes Mellitus, Non Insulin Dependent[MeSH Terms]) OR "Diabetes Mellitus, Non Insulin Dependent"[Title/Abstract]) OR Diabetes Mellitus, Non-Insulin-Dependent[MeSH Terms]) OR "Diabetes Mellitus, Non-Insulin-Dependent"[Title/Abstract]) OR Non-Insulin-Dependent Diabetes Mellitus[MeSH Terms]) OR "Non-Insulin-Dependent Diabetes Mellitus"[Title/Abstract]) OR Diabetes Mellitus, Stable[MeSH Terms]) OR "Diabetes Mellitus, Stable"[Title/Abstract]) OR Stable Diabetes Mellitus[MeSH Terms]) OR "Stable Diabetes Mellitus"[Title/Abstract]) OR Diabetes Mellitus, Type II[MeSH Terms]) OR "Diabetes Mellitus, Type II"[Title/Abstract]) OR NIDDM[MeSH Terms]) OR NIDDM[Title/Abstract]) OR Diabetes Mellitus, Noninsulin Dependent[MeSH Terms]) OR "Diabetes Mellitus, Noninsulin Dependent"[Title/Abstract]) OR Diabetes Mellitus, Maturity-Onset[MeSH Terms]) OR "Diabetes Mellitus, Maturity-Onset"[Title/Abstract]) OR Diabetes Mellitus, Maturity Onset[MeSH Terms]) OR "Diabetes Mellitus, Maturity Onset"[Title/Abstract]) OR Maturity-Onset Diabetes Mellitus[MeSH Terms]) OR "Maturity-Onset Diabetes Mellitus"[Title/Abstract]) OR Maturity Onset Diabetes Mellitus[MeSH Terms]) OR "Maturity Onset Diabetes Mellitus"[Title/Abstract]) OR MODY[MeSH Terms]) OR MODY[Title/Abstract]) OR Diabetes Mellitus, Slow-Onset[MeSH Terms]) OR "Diabetes Mellitus, Slow-Onset"[Title/Abstract]) OR Diabetes Mellitus, Slow Onset[MeSH Terms]) OR "Diabetes Mellitus, Slow Onset"[Title/Abstract]) OR Slow-Onset Diabetes Mellitus[MeSH Terms]) OR "Slow-Onset Diabetes Mellitus"[Title/Abstract]) OR Type 2 Diabetes Mellitus[MeSH Terms]) OR "Type 2 Diabetes Mellitus"[Title/Abstract]) OR Noninsulin-Dependent Diabetes Mellitus[MeSH Terms]) OR "Noninsulin-Dependent Diabetes Mellitus"[Title/Abstract]) OR Noninsulin Dependent Diabetes Mellitus[MeSH Terms]) OR "Noninsulin Dependent Diabetes Mellitus"[Title/Abstract]) OR Maturity-Onset Diabetes[MeSH Terms]) OR "Maturity-Onset Diabetes"[Title/Abstract]) OR Diabetes, Maturity-Onset[MeSH Terms]) OR "Diabetes, Maturity-Onset"[Title/Abstract]) OR Maturity Onset Diabetes[MeSH Terms]) OR "Maturity Onset Diabetes"[Title/Abstract]) OR Type 2 Diabetes[MeSH Terms]) OR "Type 2 Diabetes"[Title/Abstract]) OR Diabetes, Type 2[MeSH Terms]) OR "Diabetes, Type 2"[Title/Abstract]) OR Diabetes Mellitus, Adult-Onset[MeSH Terms]) OR "Diabetes Mellitus, Adult-Onset"[Title/Abstract]) OR Adult-Onset Diabetes Mellitus[MeSH Terms]) OR "Adult-Onset Diabetes Mellitus"[Title/Abstract]) OR Diabetes Mellitus, Adult Onset[MeSH Terms]) OR "Diabetes Mellitus, Adult Onset"[Title/Abstract])
**#5**	**#1 AND #2 AND #3 AND #4**
